# Identification of faecal *Escherichia coli* isolates with similar patterns of virulence and antimicrobial resistance genes in dogs and their owners

**DOI:** 10.1002/vms3.965

**Published:** 2022-10-12

**Authors:** Zahra Naziri, Abdollah Derakhshandeh, Sahar Zare, Malihe Akbarzadeh Niaki, Azar Motamedi Boroojeni, Vida Eraghi, Abolfazl Shirmohamadi Sosfad

**Affiliations:** ^1^ Department of Pathobiology, School of Veterinary Medicine Shiraz University Shiraz Iran

**Keywords:** dog, dog owner, *E. coli*, streptomycin resistance genes, tetracycline resistance genes, virulence gene

## Abstract

**Background:**

The presence of antimicrobial resistance and virulence genes in *Escherichia coli* allows them to survive and cause infections. The close contact between humans and pets can reinforce the risk of transmitting resistant and virulent bacteria between them.

**Objectives:**

This study aims to compare the patterns of the presence of tetracycline and streptomycin resistance genes, as well as important virulence genes in *E. coli* isolated from faeces of healthy dogs and their owners.

**Methods:**

Polymerase chain reactions were performed for detection of antimicrobial resistance (*tetA*, *tetB*, *tetC*, *tetD*, *strA* and *strB*) and virulence (*fimH*, *iss*, *sitA* and *malX*) genes in 144 faecal *E. coli* isolates from 28 dog–owner pairs and 16 humans who did not keep any pets as controls.

**Results:**

Among the investigated antimicrobial resistance and virulence genes, *tetA* (52.1%) and *fimH* (86.8%) genes had the highest prevalence. No statistically significant difference was found between the prevalence of antimicrobial resistance and virulence genes in isolates of dogs and their owners. In total, 46.4% of dog–owner pairs had the same patterns of presence or absence of six antimicrobial resistance genes, 50.0% had the same patterns of presence or absence of four virulence genes and 25.0% had the same patterns of presence or absence of all 10 tested genes.

**Conclusion:**

The presence of antimicrobial‐resistant virulent *E. coli* in humans and pets may predispose them to infections that are hard to cure with conventional antibiotics. Notable frequency of dogs’ and their owners’ *E. coli* isolates with similar patterns of antimicrobial resistance and virulence genes may indicate the possibility of sharing virulent antimicrobial resistant *E. coli* between them.

## Graphical abstract

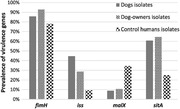

## 1 INTRODUCTION

*Escherichia coli* are well‐known commensal bacteria of the gastrointestinal tract of humans and animals, including dogs (I. Carvalho et al., 2021). As an opportunistic pathogen, *E. coli* can cause intestinal and extra‐intestinal diseases (Abreu‐Salinas et al., 2020; I. Carvalho et al., 2021). It can be shed in faeces and spread easily through feed, water and soil. Moreover, the zoonotic transmission of *E. coli* between humans and companion animals is possible (Massella et al., 2021; Qekwana et al., 2018).

*Escherichia coli* can acquire antimicrobial resistance genes from the same or different bacterial species, through horizontal gene transfer. Also, the extensive use of antibiotics in humans and animals can cause antibiotic resistance and the selection pressure on this normal flora (Skurnik et al., 2016; Yasugi et al., 2021).

In addition to the antimicrobial resistance genes, the presence of different virulence factors in *E. coli* allows them to survive and cause infections. There are several known virulence genes in *E. coli* including, adhesins such as type‐1 fimbriae (*fimH*), protectins such as increased serum survival (*iss*), sidrophores such as the iron transporter *sitA* and the pathogenicity islands marker (*malX*) with glucose and maltose transporting activity (Naziri et al., 2020).

Like humans, dogs’ intestines also can be reservoirs of commensal and pathogenic *E. coli* strains, which carry different antibiotic resistance and virulence genes. So, a human can acquire these strains through direct contact with dogs and their faeces, and also indirectly from common environments (Naziri et al., 2015). As the number of humans who keep dogs is increasing worldwide, this close contact can reinforce the risk of sharing resistant and virulent bacteria between dogs and their owners (Abreu‐Salinas et al., 2020; I. Carvalho et al., 2021). Therefore, the objective of the current study was to determine and compare the patterns of the presence of four tetracycline and two streptomycin resistance genes and also four important virulence genes in *E. coli* strains isolated from faeces of healthy dogs and their owners.

## 2 MATERIALS AND METHODS

### 2.1 Bacterial strains

A total of 144 confirmed *E. coli* strains investigated in the current study were previously isolated from faecal samples of 28 healthy dogs (two isolates from each; *n* = 56), 28 healthy dog owners (two isolates from each; *n* = 56) and 16 healthy humans who did not keep any pets as controls (two isolates from each, *n* = 32; Naziri et al., 2015). All human volunteers who participated in the sampling were over 18 years old; they filled out and signed the informed consent.

### 2.2 Antimicrobial resistance and virulence factors genes detection

In order to detect the presence of four tetracycline resistance genes (*tetA*, *tetB*, *tetC* and *tetD*) and two streptomycin resistance genes (*strA* and *strB*) and also to investigate the presence of four virulence genes including *fimH*, *iss*, *sitA* and *malX*, polymerase chain reactions (PCRs) were performed on DNA of 144 *E. coli* isolates that were extracted by the boiling method (Derakhshandeh et al., 2014). The primer sequences, amplicon size and PCR conditions were summarised in Table 1. Finally, the PCR products were electrophoresed on 1% agarose gel (Parstous) containing a safe stain (YTA) and visualised using a UV‐transilluminator (UVitec).

**TABLE 1:** Primer sequences, amplicon size and polymerase chain reaction (PCR) conditions for detection of antimicrobial resistance and virulence genes

Genes	Primer sequences (5ʹ to 3ʹ)	Amplicon size (bp)	PCR conditions	References
**Antimicrobial resistance genes**				
*tetA*	GGCCTCAATTTCCTGAC AAGCAGGATGTAGCCTGTG	372	One cycle of 3 min at 95°C; 35 cycles of 1 min at 94°C, 90 s at 57°C, 1 min at 72°C; one cycle of 10 min at 72°C	(Srinivasan et al., 2007)
*tetB*	GAGACGCAATCGAATTCG TTTAGTGGCTATTCTTCCTGC	228	Same as for *tetA* but annealing temperature was 55°C	
*tetC*	TGCTCAACGGCCTCAAC AGCAAGACGTAGCCCAGC	379	Same as for *tetB*	
*tetD*	GGATATCTCACCGCAACT CATCCATCCGGAAGTGATAG	436	Same as for *tetA* but annealing temperature was 50°C	
*strA*	CTTGGTGATAACGGCAATT CCAATCGCAGATAGAAGG	548	Same as for *tetD*	
*strB*	ATCGTCAAGGGATTGAAAC ATCGTCAAGGGATTGAAAC	509	Same as for *tetD*	
**Virulence genes**				
*fimH*	TCGAGAACGGATAAGCCGTGG GCAGTCACCTGCCCTCCGGTA	508	One cycle of 5 min at 95°C; 30 cycles of 30 s at 94°C, 30 s at 62°C, 3 min at 68°C; one cycle of 10 min at 72°C	(Rodriguez‐Siek et al., 2005)
*malX*	GGACATCCTGTTACAGCGCGCA TCGCCACCAATCACAGCCGAAC	925	Same as for *fimH*	
*sitA*	AGGGGGCACAACTGATTCTCG TACCGGGCCGTTTTCTGTGC	608	One cycle of 5 min at 95°C; 30 cycles of 30 s at 94°C, 45 s at 62°C, 90 s at 72°C; one cycle of 10 min at 72°C	
*iss*	GTGGCGAAAACTAGTAAAACAGC CGCCTCGGGGTGGATAA	760	One cycle of 4 min at 94°C; 30 cycles of 1 min at 94°C, 1 min at 58°C, 1 min at 72°C; one cycle of 5 min at 72°C	(Horne et al., 2000)

### 2.3 Statistical analysis

Comparisons of the prevalence of antimicrobial resistance and virulence genes in *E. coli* isolates of the different studied groups were performed by the Pearson chi‐square (χ2) test and Fisher's exact test (SPSS 16.0, SPSS Inc.). A value of *P* ≤ 0.05 was regarded as statistically significant.

## 3 RESULTS

### 3.1 Detection of antimicrobial resistance genes

Among 144 *E. coli* isolates, the *tetA* (52.1%) was the most prevalent antimicrobial resistance gene followed by *strA* (13.9%), *tetB* (9.7%), *tetC* (8.3%) and *strB* (7.6%). The *tetD* gene was not detected in any of *E. coli* isolates. The prevalence of tetracycline resistance genes (*tetA*, *tetB*, *tetC* and *tetD*) and streptomycin resistance genes (*strA* and *strB*) among *E. coli* isolates of three studied groups (dogs, dog owners and control humans) is shown in Table 2.

**TABLE 2:** Prevalence of antimicrobial resistance and virulence genes among *Escherichia coli* isolates of dogs, dog owners and control humans^a^

	Dogs			Dog owners			Control humans		
	All (*n* = 56)	Male (*n* = 28)	Female (*n* = 28)	All (*n* = 56)	Male (*n* = 30)	Female (*n* = 26)	All (*n* = 32)	Male (*n* = 18)	Female (*n* = 14)
**Prevalence of antimicrobial resistance genes**									
***tetA***	33 (58.9)	16 (57.1)	17 (60.7)	27 (48.2)	15 (50.0)	12 (46.2)	15 (46.9)	10 (55.6)	5 (35.7)
***tetB***	5 (8.9)	3 (10.7)	2 (7.1)	5 (8.9)	4 (13.3)	1 (3.8)	4 (12.5)	0 (0.0)	4 (28.6)
***tetC***	5 (8.9)	2 (7.1)	3 (10.7)	5 (8.9)	3 (10.0)	2 (7.7)	2 (6.2)	0 (0.0)	2 (14.3)
***tetD***	0 (0.0)	0 (0.0)	0 (0.0)	0 (0.0)	0 (0.0)	0 (0.0)	0 (0.0)	0 (0.0)	0 (0.0)
***strA***	8 (14.3)	4 (14.3)	4 (14.3)	5 (8.9)	5 (16.7)	0 (0.0)	7 (21.9)	3 (16.7)	4 (28.6)
***strB***	7 (12.5)	2 (7.1)	5 (17.9)	1 (1.8)	1 (3.3)	0 (0.0)	3 (9.4)	1 (5.6)	2 (14.3)
**Prevalence of virulence genes**									
***fimH***	48 (85.7)	27 (96.4)	21 (75.0)	52 (92.9)	27 (90.0)	25 (96.2)	25 (78.1)	16 (88.9)	9 (64.3)
***iss***	25 (44.6)	10 (35.7)	15 (53.6)	16 (28.6)	12 (40.0)	4 (15.4)	3 (9.4)	0 (0.0)	3 (21.4)
***malX***	5 (8.9)	2 (7.1)	3 (10.7)	6 (10.7)	4 (13.3)	2 (7.7)	11 (34.4)	7 (38.9)	4 (28.6)
***sitA***	34 (60.7)	16 (57.1)	18 (64.3)	36 (64.3)	20 (66.7)	16 (61.5)	8 (25.0)	4 (22.2)	4 (28.6)

^a^ Values are shown as number (%).

The prevalence of four tetracycline and two streptomycin resistance genes was not significantly different in isolates of dogs, dog owners and control humans (*P* > 0.05). Moreover, no significant difference (*P* > 0.05) was found in the prevalence of these resistance genes between females and males isolates in each groups, except for the *tetB* gene, which was more prevalent (*P* = 0.028) in female control humans isolates, and the *strA* gene, which was more prevalent in male owners isolates (*P* = 0.055).

**FIGURE 1:**
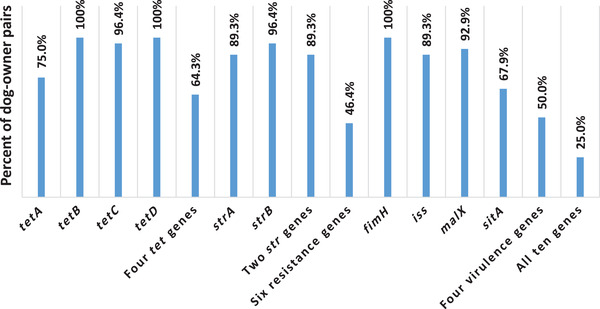
Frequencies of dog–owner pairs who had *Escherichia coli* isolates with a similar status of presence or absence of antimicrobial resistance and virulence genes

Comparison of patterns of the antimicrobial resistance genes in *E. coli* isolates of dogs and their owners revealed that among 28 dog–owner pairs, in 21, 28, 27 and 28 pairs, the status of presence or absence of the *tetA*, *tetB*, *tetC* and *tetD* genes was similar in at least one *E. coli* isolate of dogs and their owners. In 18 pairs, the patterns of presence or absence of all the four tested tetracycline resistance genes were similar in at least one *E. coli* isolate of dogs and their owners. Furthermore, 25 and 27 dog–owner pairs had at least one *E. coli* isolate with a similar status of presence or absence of the *strA* and *strB* genes. In 25 pairs, the patterns of presence or absence of the both tested streptomycin resistance genes were similar in at least one *E. coli* isolate of dogs and their owners. Among 28 dog–owner pairs, at least one *E. coli* isolate of 13 pairs had the same patterns of presence or absence of all the six tested antimicrobial resistance genes (Figure 1).

### 3.2 Detection of virulence genes

Among 144 *E. coli* isolates, the *fimH* (86.8%) was the most prevalent virulence gene, followed by *sitA* (54.2%), *iss* (30.6%) and *malX* (15.3%) genes. The prevalence of four virulence genes (*fimH*, *iss*, *malX* and *sitA*) among the *E. coli* isolates of three studied groups (dogs, dog owners and control humans) is shown in Table 2.

**FIGURE 2:**
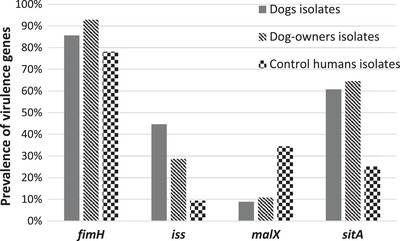
Comparison of the prevalence of four virulence genes in *E. coli* isolates of dogs, dog owners and control humans

Statistical analysis revealed that the prevalence of four studied virulence genes was not significantly different in isolates of dogs and their owners (*P* > 0.05). The *iss* and *sitA* genes were significantly more prevalent in the *E. coli* isolates of dogs and dog owners than in control humans’ (*P* < 0.05). Only the prevalence of *malX* gene was significantly higher in control humans’ isolates than dogs’ (*P* = 0.003) and dog owners’ (*P* = 0.007) isolates (Figure 2).

Moreover, no significant difference (*P* > 0.05) was found in the prevalence of these four virulence genes between females’ and males’ isolates in each groups, except for the *fimH* gene, which was more prevalent (*P* = 0.051) in male dogs’ isolates, and the *iss* gene, which was more prevalent in female owners’ isolates (*P* = 0.042).

Comparison of patterns of the virulence genes in *E. coli* isolates of dogs and their owners revealed that among 28 dog–owner pairs, at least one *E. coli* isolate from 28, 25, 26 and 19 pairs were similar in the status of presence or absence of the *fimH*, *iss*, *malX* and *sitA* genes. In 14 pairs, the patterns of presence or absence of all the four tested virulence genes were similar in at least one *E. coli* isolate of dogs and their owners (Figure 1).

It is noteworthy that at least one *E. coli* isolate of seven (25.0%) dog–owner pairs had the same patterns of presence or absence of all the 10 tested virulence genes and antimicrobial resistance genes (Figure 1).

## 4 DISCUSSION

Similar patterns of presence or absence of all the six tested antimicrobial resistance genes in the *E. coli* isolates of 46.4% of dog–owner pairs, similar patterns of presence or absence of all the four tested virulence genes in the *E. coli* isolates of 50.0% of dog–owner pairs, and finally similar patterns of presence or absence of all the 10 tested antimicrobial resistance and virulence genes in the *E. coli* isolates of 25.0% of dog–owner pairs may indicate the possibility of sharing the virulent antimicrobial resistant *E. coli* between dogs and their owners.

In our previous research on these *E. coli* isolates, the highest frequency of antimicrobial resistance was observed against streptomycin (41.7%) and tetracycline (38.9%) using the disk diffusion technique (Clinical & Laboratory Standards Institute, 2018). In detail, the frequencies of streptomycin‐resistant *E. coli* were 48.2%, 37.5% and 37.5% in dogs, owners and control humans, respectively. Moreover, the frequencies of tetracycline‐resistant *E. coli* were 53.6%, 30.4% and 28.1% in dogs, owners and control humans, respectively (Naziri et al., 2022).

In I. Carvalho et al.’s (2021) study, a significant prevalence of *tetA* or *tetB* genes was found among most of the tetracycline resistant *E. coli* isolates. In their study, the prevalence of tetracycline‐resistant *E. coli* was 80% and 90% in healthy and sick dogs, respectively. Furthermore, the prevalence of streptomycin‐resistant *E. coli* was 60% and 40% in healthy and sick dogs, respectively (I. Carvalho et al., 2021).

Karahutová et al. (2021) stated that healthy dogs can be a potential reservoir of resistant bacteria. In their study, antimicrobial resistance genes were more frequent in *E. coli* isolates of healthy dogs than in diarrhoeic dogs. The highest frequency of antimicrobial resistance in faecal *E. coli* isolates of both healthy and diarrhoeic dogs was against tetracycline (34.21% and 31.11%, respectively). The *tetA* gene (50%) was more prevalent in healthy dogs, while the *tetB* gene was only detected in 13.18% of the *E. coli* isolates from healthy dogs. On the contrary, in diarrhoeic dogs, the prevalence of *tetB* gene (28.89%) was higher than *tetA* gene (11.11%; Karahutová et al., 2021).

In a similar study conducted by A. C. Carvalho et al. (2016) on the *E. coli* isolates from dogs and their owners, the prevalence of streptomycin and tetracycline resistance in dogs’ isolates was 66.6% and 50.0%, respectively. Moreover, the prevalence of streptomycin and tetracycline resistance in owners’ isolates was 64.3% and 52.3%, respectively. In their study, the highest frequency of resistance was recorded against ampicillin, tetracycline, streptomycin and trimethoprim‐sulfamethoxazole (A. C. Carvalho et al., 2016).

Furthermore, there were other comparable studies on the prevalence of streptomycin and tetracycline resistance genes in *E. coli* isolates from human and companion animals (Chung et al., 2017; Poudel et al., 2019; Skurnik et al., 2016; Toombs‐Ruane et al., 2020; Yasugi et al., 2021).

Besides the antimicrobial resistance genes that have an important role in the survival of bacteria, the virulence genes also have a key role in the pathogenesis of bacteria (Massella et al., 2021). *Escherichia coli* have more than 50 genes that encode adhesins, toxins, siderophores, invasins, protectin, capsular antigens and miscellaneous virulence genes that give them the ability of attaching and colonising the host cells, acquisition of iron, toxicity, pathogenicity and so forth (Flament‐Simon et al., 2020; Naziri et al., 2020).

In the present research, the *fimH* virulence gene had the highest prevalence in *E. coli* isolates of humans (87.5%) and dogs (85.7%). In line with the present research, in a study by Bahadori et al. (2019), the *fimH* gene was the most frequent (62.2%) virulence gene in human faecal *E. coli* isolates (Bahadori et al., 2019). Moreover, prevalence of 100% (Johnson et al., 2001), 97.5% (Flament‐Simon et al., 2020), 86.2% (Valat et al., 2020) and 80% (Johnson et al., 2009) was reported for the *fimH* gene in dog's *E. coli* isolates.

The prevalence of *malX* gene in *E. coli* isolates of humans and dogs was 19.3% and 8.9%, respectively. In a study by Bahadori et al. (2019), the prevalence of *malX* gene in human faecal *E. coli* isolates was 27.7% (Bahadori et al., 2019). Moreover, prevalence of 20% (Johnson et al., 2009) and 47.2% (Flament‐Simon et al., 2020) was reported for the *malX* gene in dog's *E. coli* isolates.

In the present research, the prevalence of *sitA* gene in *E. coli* isolates of dogs and humans was 60.7% and 50.0%, respectively. This prevalence was 87.5% (Yasugi et al., 2021), 79% (Vangchhia et al., 2016) and 62.5% (Ewers et al., 2010) in previous studies. Furthermore, we detected the *iss* gene in 44.6% of dog's faecal *E. coli* isolates. This prevalence was 10% (Johnson et al., 2009), 15.5% (Valat et al., 2020), 15.7% (Flament‐Simon et al., 2020) and 100% (Yasugi et al., 2021) in previous research.

Variable frequencies of virulence factors and antimicrobial resistance genes can be due to differences in strains of studied *E. coli* in various geographical regions.

## 5 CONCLUSION

The presence of antimicrobial‐resistant virulent *E. coli* in humans and pets may predispose them to infectious diseases that are hard to cure with conventional antibiotics. Moreover, the notable frequency of *E. coli* isolates with similar antimicrobial resistance and virulence genes patterns in dogs and their owners may indicate the possibility of sharing the virulent antimicrobial‐resistant *E. coli* between them.

## AUTHOR CONTRIBUTIONS

*Study design and acquisition of funding*: Abdollah Derakhshandeh. *Methodology*: Sahar Zare, Malihe Akbarzadeh Niaki, Azar Motamedi Boroojeni and Vida Eraghi, Zahra Naziri. *Analysis and interpretation of data*: Abolfazl Shirmohamadi Sosfad and Zahra Naziri. *Drafting the manuscript*: Abolfazl Shirmohamadi Sosfad and Zahra Naziri. *Editing and writing the original article*: Zahra Naziri. All authors read and approved the final version of the manuscript.

### PEER REVIEW

The peer review history for this article is available at https://publons.com/publon/10.1002/vms3.965.

## ETHICS STATEMENT

The authors confirm that the ethical policies of the journal, as noted on the journal's author guidelines page, have been adhered to, and this study was performed as stated by the Declaration of Helsinki principles. The Ethics Committee of the School of Veterinary Medicine, Shiraz University, certified all protocols (Register numbers: PHD891925). All volunteer human participants signed an informed consent.

## Data Availability

Further data are available from the corresponding author upon reasonable request.
